# *Amaranthus graecizans* L. Mitigates Hyperlipidemia-Induced Nonalcoholic Fatty Liver Disease in Experimental Rats: Future Pharmaceuticals

**DOI:** 10.3390/ph18081196

**Published:** 2025-08-13

**Authors:** Nadiah S. Alzahrani, Bayan Aljahdali, Aeshah Alhosain, Abeer Abdullah Alasmari, Touseef Amna, Soha Mohamed Yousef

**Affiliations:** 1 College of Science, Al-Baha University, Al Baha 65779, Saudi Arabia; 2 Department of Home Economics, College of Education, Najran University, Najran 11001, Saudi Arabia; baaljahdali@nu.edu.sa; 3 Department of Food Science and Human Nutrition, Qassim University, Buraydah 52571, Saudi Arabia; abhsairn@qu.edu.sa; 4 Department of Nutrition and Food Science, King Saud University, Riyadh 11451, Saudi Arabia; 439203421@student.ksu.edu.sa; 5 Department of Biology, Faculty of Science, Al-Baha University, Al-Baha 65779, Saudi Arabia; 6 Department of Home Economics, Faculty of Specific Education, Fayoum University, Fayoum 63514, Egypt; smy01@fayoum.edu.eg; 7 Applied College in Almakhwah, Al-Baha University, Al-Baha 65779, Saudi Arabia

**Keywords:** proximate analysis, lipid profiles, liver function, histological examination

## Abstract

**Background:** Nonalcoholic fatty liver disease (NAFLD) associated with hyperlipidemia is a prevalent metabolic disorder, often triggered by high-fat diets (HFDs) in animal models. *Amaranthus graecizans* L. (AGs), rich in bioactive compounds, offers potential antioxidant and lipid-lowering benefits, making it a candidate for natural liver protection. This study evaluated the protective role of *Amaranthus graecizans* L. against hyperlipidemia-induced NAFLD in rats. **Methods:** Thirty male Wistar rats (150 ± 20 g, 10 weeks old) were split into five groups (*n* = 6 each). A control group received 0.25 mL 0.1% DMSO orally. Four HFD-fed groups included one with only DMSO (0.25 mL) and three supplemented with AG solution (0.25 mL) at 100, 200, or 500 mg/kg body weight. Treatments were given daily via gavage for two months. AGs’ nutritional profile, serum lipids, liver function, and liver histology were analyzed. **Results:** AGs contain 21.3% protein, 1.1% fat, 15% fiber, moderate vitamins (ascorbic acid, B-complex), and minerals (high potassium, calcium; low magnesium, phosphorus, sodium). AG-treated rats weighed less than the HFD controls. Unlike the control group (normal lipids, liver function, no steatosis), the HFD rats showed severe hyperlipidemia, liver dysfunction, and steatosis with fat changes. The AG groups exhibited dose-dependent improvements in lipids and liver function; the 200 mg/kg group had reduced fatty changes, and the 500 mg/kg group showed minimal hepatocyte fat. **Conclusions:** *Amaranthus graecizans* L. reduces hyperlipidemia and NAFLD progression in HFD-fed rats, which suggests its potential as a natural liver-protective agent.

## 1. Introduction

Nonalcoholic fatty liver disease involves the excessive accumulation of fat in the liver. Important to point out is that, NAFLD is a serious health problem since it may evolve from simple fat accumulation to severely advanced stages. The evolution of NAFLD includes more severe and potentially progressive forms, including the disease that we do and do not consider a form of alcoholic liver disease: nonalcoholic steatohepatitis (NASH). So, when NAFLD becomes more severe, it can lead to fibrosis and cirrhosis, which can be a major problem in public health whenever left untreated [1,2]. The prevalence of NAFLD has significantly increased globally and it affects about 30.05% of the global population [3,4]. NAFLD contributes to advanced liver disease necessitating transplantation and is a key factor in liver-related mortality [2]. It is becoming almost like an epidemic with the rise of type 2 diabetes and obesity [5]. By 2030, NAFLD will probably become the foremost cause of liver transplantation. A more prevalent history of cases also puts the statistics of NAFLD in the Middle East very much on the increase, increasing from 36.53% in 1990 to 42.62% in 2019 [6]. A comprehensive analysis found that NAFLD affects about 16.8% of the population of Saudi Arabia. Obesity rates in the country exceed global norms, likely contributing to the elevated occurrence of NAFLD in this area [7].

Metabolic syndrome (MS) adds to the peril of increased risk of NAFLD. Insulin resistance appears to be the main mechanism behind these above-mentioned processes according to some previous studies [8,9]. The components of metabolic syndrome include systemic hypertension, dyslipidemia, and insulin resistance [9]. Clinical management for metabolic syndrome includes lifestyle modifications such as dietary changes that promote decreased caloric intake; increasing physical activity; and pharmacotherapy aimed at decreasing the risks of dyslipidemia, hyperglycemia, hypertension, and increased LDL-C and decreased HDL-C levels, as well as triglycerides [8,10]. In such cases, functional foods mainly from fruits and vegetables are highly recommended due to their abundance in bioactive compounds, predominantly polyphenols [10,11].

*Amaranthus graecizans* L. (AGs) is a plant that belongs to the Amaranthaceae family. Originally from the Mediterranean region, this herbaceous plant has now expanded to other parts of the world, including Africa, Asia, and America [12]. AG is consumed as a leafy vegetable in Africa and Asia, whether boiled or stir-fried. Its leaves are high in minerals including iron, calcium, and vitamins, making it an important supplement to local diets, especially in rural regions. The seeds, while less widely utilized, are occasionally collected for their edible grains, which are high in protein and fiber [13]. Recent studies have focused on identifying the therapeutic ingredients of AG [12,13]. Extraction from all parts of AG is expected to possess medicinal properties. Research has been intensified in recent years regarding the nutraceutical and therapeutic possible use of Amaranthus species as functional foods owing to their high antioxidant activity and anti-inflammatory properties [14]. Phytochemical screening of the aerial parts of some AG species has provided evidence of the presence of bioactive components such as steroids, glycosides, alkaloids, phenolic acids, flavonoids, amino acids, betaine, lipids, carotenoids, and catechuic tannins [15].

A review study indicated that these polyphenols could mitigate the effects of obesity and several metabolic problems caused by obesity, such as hypertension, glucose intolerance, and dyslipidemia, while also providing protection against organ damage [14]. The pharmacological effect exhibited by AGs can be attributed to numerous bioactive phytoconstituents as discovered in previous studies [15,16,17]. A polyphenol-rich diet, Amaranthus being included, seems to provide reasonable protection against several chronic diseases [18]. Dutta and Singh [19] studied the effects of AG leaf extraction on genomic DNA and lipid peroxidation in goat liver, with the findings indicating the significant enhancement in antioxidant levels. The results suggest that elevated antioxidant levels counteract oxidative damage, potentially mitigating degenerative conditions linked to metabolic stress, genotoxicity, and cytotoxicity. However, limited research exists on the protective effects of AGs against hyperlipidemia-induced NAFLD, though plants in the Amaranthaceae family are known for various health and medicinal properties. This highlights the need for in-depth studies to assess AGs’ value as a functional food and therapeutic agent. The current investigation examined AGs’ ability to safeguard rats from hyperlipidemia-induced NAFLD.

## 2. Results

### 2.1. Proximate Composition of Amaranthus graecizans *L.*

Table 1 shows the results of the proximate composition of AG plants. The carbohydrate content of AGs was 31.5%. The AGs have a significant amount of protein (21.3%), while the total fat was 1.1%. The moisture and ash contents in the AGs were 5.15% and 25.6%, respectively. In addition, it was discovered that the AGs contained a high fiber content of (15%).

### 2.2. Vitamin and Mineral Content of Amaranthus graecizans *L.*

The vitamin and mineral contents of AGs are presented in Table 2. The results outlined that the AGs’ powder contained a moderate amount of ascorbic acid and B-complex vitamins (Niacin B3, Thiamine B1, Cobalamin B12, Folic acid B9). In addition, a moderate amount of vitamin A, vitamin E, and vitamin D3 was found in the AGs. The results indicated that AGs’ powder contains high amounts of potassium and calcium. However, it contains small amounts of magnesium, phosphorus, and sodium.

### 2.3. Phenolic Compounds of Amaranthus graecizans *L.*

The result presented in Table 3 shows the phenolic compounds of AGs with their retention times, molecular weights, molecular formulae, and peak area percentages of each compound. The major compound found in AGs was chlorogenic acid (21.20%). The compound with the second largest peak area percentage was gallic acid (16.96%). Some other phenolic compounds were recognized such as rutin (8.71%), rosmarinic acid (6.52%), Vanillin (4.81), Caffeic acid (3.21%), Cinnamic acid (3.01%), p-Coumaric acid (2.69%), Methyl gallate (2.47%), Ferulic acid (2.3%), and Syringic acid (1.22%) as well as catechin and Daidzein (0.59%, 0.54%), respectively (see File S1 in the Supplementary Materials).

### 2.4. Evaluation of the Body Weight Gain

Rats on an HFD exhibited a notably greater body weight increase than those on a standard diet (SD) (Table 4). In contrast, the HFD-fed rats treated with AGs at doses of 100, 200, and 500 mg/kg showed a substantial decrease in weight gain compared to the untreated HFD group.

### 2.5. Effect of AGs on Serum Lipid Profiles

**Figure 1:**
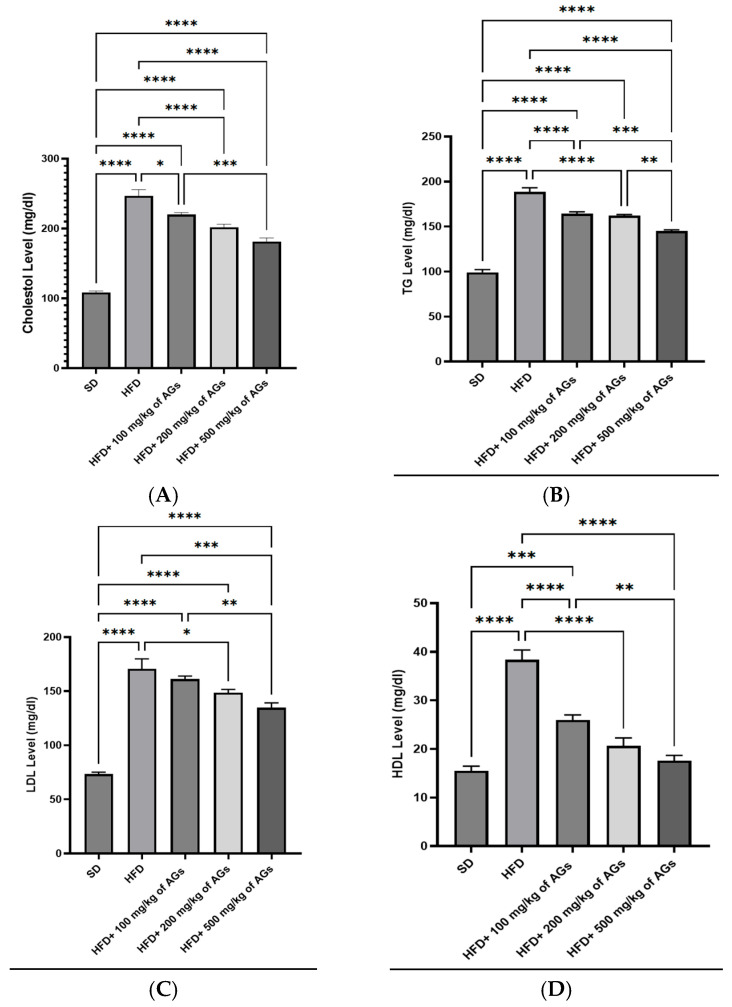
Effect of AG dose-dependent treatment on the following: (**A**) cholesterol levels; (**B**) TG, triglyceride levels; (**C**) LDL-c, low-density lipoprotein cholesterol levels; (**D**) HDL-c, high-density lipoprotein cholesterol levels. The bracket represents statistically significant differences among groups at * *p*-value < 0.05; ** *p*-value < 0.005; *** *p*-value < 0.0005; and **** *p*-value < 0.0001.

The outcome demonstrated that after 2 months of high-fat diets, the serum levels of lipid profiles were significantly increased in the HFD groups as compared to the controls (Figure 1A–D). It was found that there was a very significant decrease (*p* < 0.0001) in serum total cholesterol in both the HFD + 200 mg/kg of AG and HFD + 500 mg/kg of AG groups, compared to the HFD group. In addition, all tested doses of AGs (100, 200, 500 mg/kg) decreased the serum TG levels significantly, compared to the HFD groups, with the best result in the HFD + 500 mg/kg of AG group relative to the HFD group concerning the LDL-c levels.

### 2.6. Effect of AGs on Liver Function

**Figure 2:**
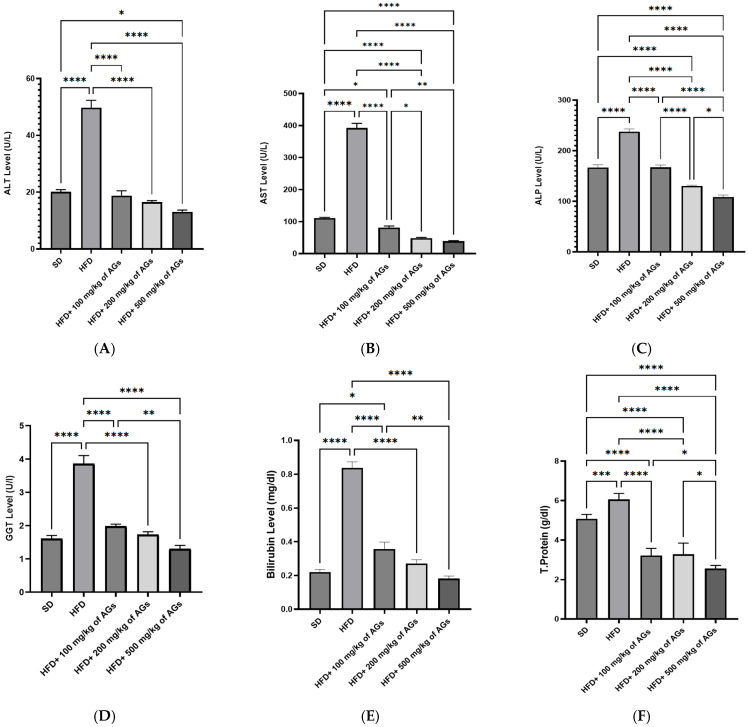
Effect of AG dose-dependent treatment on (**A**) ALT, alanine aminotransferase levels; (**B**) AST, aspartate aminotransferase levels; (**C**) ALP, alkaline phosphatase levels; (**D**) GGT, gamma-glutamyle transferase levels; (**E**) bilirubin levels; (**F**) total protein levels. The bracket represents statistically significant differences among groups at * *p*-value < 0.05; ** *p*-value < 0.005; *** *p*-value < 0.0005; and **** *p*-value < 0.0001.

HFD rats that were untreated had elevated serum levels of liver enzymes (ALT, AST, and ALP) compared to the control (Figure 2A–C). Nevertheless, the liver enzymes significantly improved in the HFD-fed animals treated with 100, 200, and 500 mg/kg AGs when compared to HFD alone. Furthermore, the results showed significant increases in the GGT, bilirubin, and total protein levels in the untreated HFD rats as compared to the control rats on standard diet (Figure 2D–F). Yet, there was a significant decrease in these parameters in the HFD rats receiving all tested doses (100, 200, and 500 mg/kg AGs) when compared to HFD alone.

### 2.7. Histological Examination

**Figure 3:**
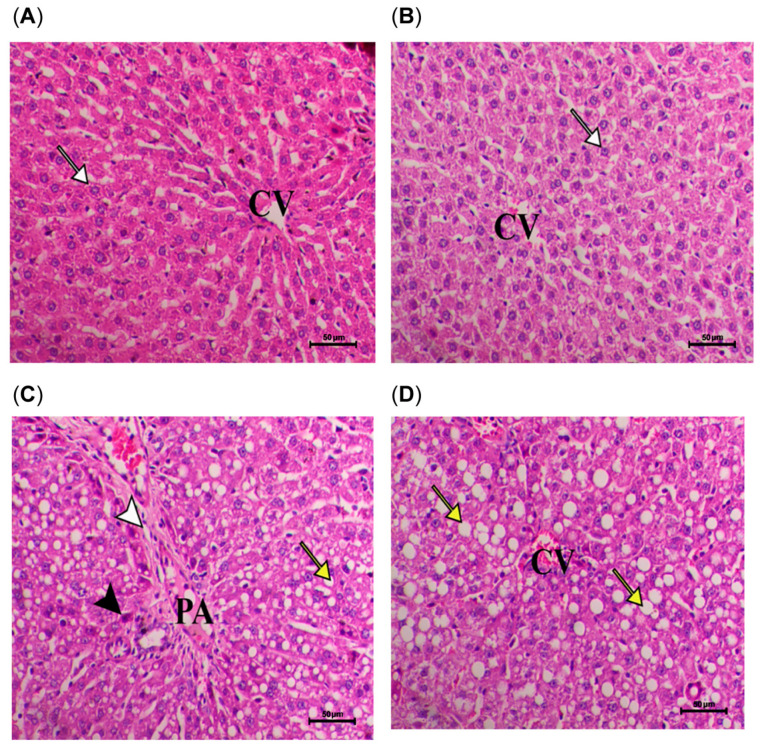
Histological examination of liver for SD and HFD groups of rats. (**A**,**B**) Liver of control animal showing normal hepatic parenchyma consisted of normal hepatocytes (white arrow) arranged in cords around the central vein (CV). (**C**) Liver of HFD animal showing marked degree of periportal hepatic vacuolar changes consistent with severe fatty changes (yellow arrow) associated with increased apoptosis of hepatocytes (black arrowhead) and periportal fibrosis (white arrowhead) (PA portal area). (**D**) Liver of HFD animal showing marked degree of diffuse lobular hepatic fatty changes and changes associated with the presence of large clear vacuoles (yellow arrows).

**Figure 4:**
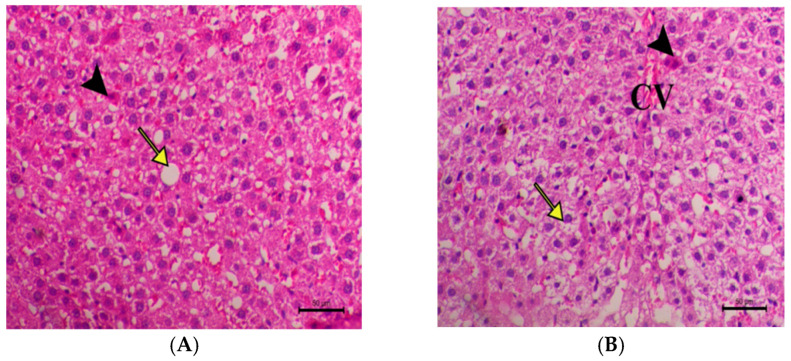

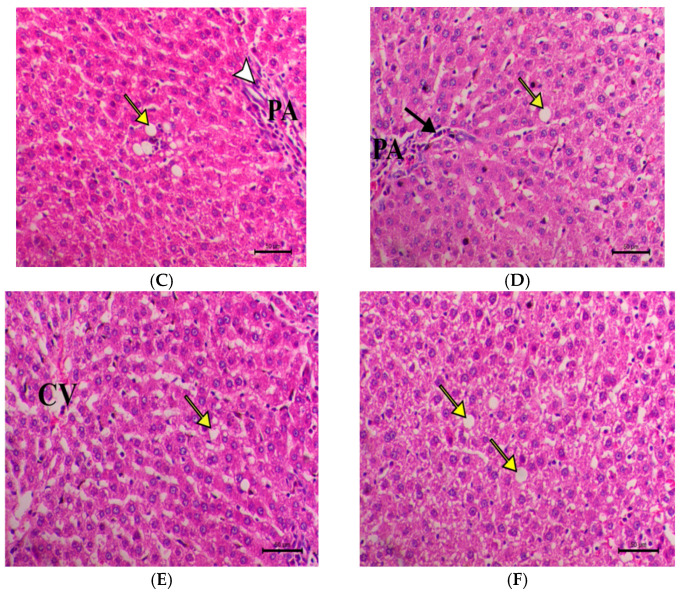
Histological examination of liver for treated groups of rats. (**A**,**B**) Liver of HFD + 100 mg/kg of AGs showing decrease in the hepatic vacuolar changes within the cytoplasm of the hepatocytes (yellow arrow reveals decrease fat vacuoles) and also a decrease in hepatic apoptotic changes (black arrowhead) (CV central vein). (**C**) Liver of HFD + 200 mg/kg of AGs showing focal hepatic fatty changes (yellow arrow) with mild periportal fibrosis (white arrowhead) (PA indicates portal area). (**D**) Liver of HFD + 200 mg /kg of AGs showing noticeable decrease in the hepatic fatty changes (yellow arrow) with mild periportal infiltration of inflammatory cells (black arrow) (PA indicates portal area). (**E**,**F**) Liver of HFD + 500 mg/kg of AGs showing marked decrease in hepatic fatty accumulation within the hepatocytes, which is seen with few hepatocytes (yellow arrow).

Histological examinations of the liver tissues are shown in Figure 3 and Figure 4. In the control group (SD), the liver tissue displayed a typical structure, with healthy hepatocytes organized in cords radiating from the central vein and separated by blood sinusoids. In contrast, the HFD group exhibited significant periportal vacuolation, indicative of severe fatty degeneration, along with heightened hepatocyte apoptosis and periportal fibrosis. Moreover, marked degrees of diffuse lobular hepatic fatty changes and changes associated with the presence of large clear vacuoles were shown in the same group. Meanwhile, a decrease in the hepatic vacuolar changes within the cytoplasm of the hepatocytes and a decrease in hepatic apoptotic changes were obvious in the (HFD + 100 mg/kg of AG) group. The same group showed a decrease in the hepatic fatty changes within the cytoplasm of the hepatocytes and also a decrease in the hepatic apoptotic changes. As for the rats of the (HFD + 200 mg/kg of AG) group, liver examination showed focal hepatic fatty changes and with mild periportal fibrosis, a noticeable decrease in the hepatic fatty changes with mild periportal infiltration of inflammatory cells. Regarding the liver examination of the (HFD + 500 mg/kg of AG) group, it showed a marked decrease in hepatic fatty accumulation within the hepatocytes, which are seen with few hepatocytes; while the remaining hepatocytes are within the normal range, a marked decrease in hepatic fatty changes within the hepatocytes is seen. The improvement in liver tissues was noticeable in the AG groups.

## 3. Discussion

One of the most important organs for metabolic processes such as cholesterol metabolism, gluconeogenesis, and de novo lipogenesis is the liver. The high prevalence of obesity and metabolic syndrome contributes to pathophysiological changes that can cause NAFLD. The recent increases in NAFLD prevalence have become a global health concern [20]. Hyperlipidemia increases in lipogenesis in the liver and steatosis are typical features of HFD-induced NAFLD in experimental rats [21]. It is found that chronic HFD feeding is the most effective means to induce obesity, metabolic abnormalities, and NAFLD in rats [22]. Plants have been widely used to avoid, treat, or even prevent the progression of many ailments such as cancer, cardiovascular, metabolic, and autoimmune diseases owing to their innumerable biological effects such as antioxidant, anti-inflammatory, antimicrobial, and antimutagenic activities [23].

In this study, we propose a dose-dependent treatment strategy for HFD-induced hepatic steatosis in experimental rats by administering different doses of AG plants. In addition, we are establishing that these positive effects are mediated, at least, by hypolipidemic and antioxidant potentials. Obesity was induced by a typical tactic of chronically feeding the experiment rats a high-fat diet [24]. Obesity complications include IR, hypertension, hyperglycemia, and hyperinsulinemia, which may have an adverse effect on general health and accelerate liver damage [25].

The chemical composition of AGs was analyzed to reveal its bioactive compounds. Approximate analyses of AGs were conducted to detect the quantities of carbohydrates, protein, fat, moisture, ash, and total fiber by using different standard methods. The proximate composition of Saudi AGs (Table 2) differs in relativity compared to the African plants, which were found to contain carbohydrate (0%), protein (2.85%), fat (0%), ash (2.20%), and moisture (82%) [26]. The chemical composition of the same plant species at the same stage of growth varies greatly depending on the environment, even if the surrounding is close by. Koutsoukis et al. (2019) explained that could be due to the impact of environmental factors such as climate and ecological concerns [27,28]. In addition, AG plants are composed of many vitamins such as B1, B3, B9, B12, C, A, E, and D3. Also, many crucial minerals were identified in AG plants including Mg, K, P, Na, and Ca (Table 3). They are almost similar to the African AG [26], except for a noticeable difference in the percentage of both vitamin C and E in favor of the Saudi AGs. AGs are nutrient-dense plants that have several advantages for liver health because of their high vitamin, mineral, and antioxidant content. The vitamin A and C content in AGs supports detoxification procedures and shields liver cells from oxidative damage. Additionally, the plant is a robust source of vitamin B, which is vital for metabolism and liver cell regeneration. Furthermore, by boosting enzymatic activity, lowering inflammation, and encouraging detoxification, calcium and magnesium included in AGs are essential for preserving the liver function. This plant is a viable option for promoting liver health and reducing liver-related illnesses because of the combination of these components [14,29,30].

Phenolic compounds, the bioactive compounds in AG’s plant, have been identified by HPLC, having 13 polyphenolic compounds, the same number that is also reported by Ishtiaq et al. (2014) and presented in Table 4 [31]. The major compound in the plant is chlorogenic acid (21.20%), which has antioxidants and anti-inflammatory actions [32]. The compound with the next largest peak area percentage was gallic acid (16.96%). Some more phenolic compounds were recognized; these phenolic compounds are in turn protective against several health issues due to their biological activities like antioxidant, antiulcer, anti-inflammatory, hypolipidemic, anti-diabetic, and antitumor [33].

In addition, in this study, the condition known as dyslipidemia was induced by an HFD, characterized by an increase in the circulating levels of LDL-c and TG [34]. On the contrary, the different dosages of AGs inhibited the dose-dependent elevation of lipid levels against anti-hyperlipidemic action. This activity may result from the high acid content of chlorogenic acid and gallic acid, which are both abundantly sourced from this plant, and which lowers the lipid levels via a decreased biosynthesis of LDL-c by the inhibition of 3-hydroxy-3-methylglutaryl-CoA (HMG-CoA), and by decreasing the intestinal absorption of lipids [35,36].

According to further supporting data, chlorogenic acid efficiently lowers fat buildup and enhances lipid metabolism by modifying important enzymes and genes involved in cholesterol homeostasis [37]. Gallic acid’s lipid-lowering properties are also attributed to its strong antioxidant and α-glucosidase inhibitory properties. Improved hypolipidemic potential has been associated with increased gallic acid bioactivity via enzymatic transformation [38]. The combined results demonstrate AGs’ potential as a natural treatment for dyslipidemia, with the synergistic effects of gallic and chlorogenic acids most likely mediating their benefits.

Additionally, the increase in liver enzyme levels points to the action of an HFD in increasing oxidative stress in rats, something mostly affirmed by many earlier studies [39,40]. But, indeed, oxidative stress was ameliorated in the HFD-fed groups supplemented with different doses of AGs in a dose-dependent manner; AGs’ seed thus holds a virtuous promise of possible antioxidant activity. Our results are in the same line with the study performed by Saha et al., which emphasized phenolics’ role in hepatoprotection against oxidative damage, thereby helping in the stabilization of cell membrane networks and inhibiting the release and expression of several proinflammatory cytokines such as tumor necrosis factor alpha (TNF-α), transforming growth factor beta (TGF-β), and various interleukins (IL-6, IL-2, and IL-8) [41].

*Amaranthus viridis* methanol extracts, for example, have demonstrated notable scavenging activities that demonstrate their efficacy as natural antioxidants and possible hepatoprotective agents, highlighting their antioxidant potential [42]. Further highlighting the therapeutic efficacy of Amaranthus species, ethanolic leaf extracts from *Amaranthus gangeticus* showed hepatoprotective benefits through polyphenolic activity [43]. It is well recognized that the phenolic chemicals in AGs, such as gallic acid and chlorogenic acid, are essential for lowering inflammation and oxidative stress. These substances aid in cell membrane stabilization and inhibit the synthesis of interleukins IL-6, IL-2, and IL-8, as well as inflammatory cytokines including TNF-α and TGF-β [41]. Additionally, a study of Amaranthus species highlights the hepatoprotective potential of phenolic components and supports their antioxidant abilities [44]. Further support is provided by research on similar phenolic-rich extracts, such as *Gentiana scabra*, which considerably decreased oxidative stress and liver damage by activating polyphenols like Quercetin and Kaempfero [45]. These results are consistent with the benefits perceived with AGs, pointing to a similar mechanism including anti-inflammatory and free radical scavenging qualities. The combined results show that AGs are viable natural remedies for liver health and oxidative stress management. Its phenolic components are essential for lowering liver enzyme levels and preventing oxidative damage and inflammation.

Several studies have suggested that the serum level of bilirubin is inversely linked with many health conditions such as obesity, metabolic syndrome, T2D, and oxidative stress-mediated diseases due to the increase in the glucuronic UGT1A1 enzyme in the liver among obese subjects that can clear bilirubin from the blood [46,47]. However, a significant increase in bilirubin levels was found among rats fed an HFD, in a similar study performed by Fu et al. (2021), which predicted that subjects would be metabolically healthy [48]. The significant decrease in bilirubin levels was noticed in groups that were fed different doses of AGs, suggesting that it may have bilirubin-lowering ability due to its phenolic compounds and that may occur via the upregulation of hepatic constitutive androstane receptor (CAR) as well as cytochrome P3A1 (CYP3A1) that can facilitate the clearance of bilirubin [49]. AGs’ phenolic components may help decrease bilirubin by triggering important liver pathways. By upregulating enzymes such as CYP3A1 and CYP2A5, studies demonstrate how the constitutive androstane receptor (CAR) improves bilirubin elimination [50,51]. Similar effects have been seen by compounds like 6,7-dimethylesculetin, confirming the possible mechanism of AG. Furthermore, bilirubin’s connection to enzymatic transcription is highlighted by its control of CYP1A1 through the aryl hydrocarbon receptor (AHR) [52]. These results imply that the phenolic chemicals in AGs may enhance metabolic health, lower oxidative stress, and improve bilirubin metabolism. Alleviated levels of total serum protein were noticed in rats fed an HFD, which are similar to the results of a previous study. Gabuza et al. (2020) suggested an association between the increased serum level of T protein [53] and obesity-related diseases. On the other hand, phenolic compounds such as chlorogenic acid, catechin, and rutin can bind to serum protein such as albumin [54].

The most crucial observation in this study is the ability of our intervention to reverse hepatic steatosis combined with lowering the lipid levels in the HFD-treated rats. However, an HFD causes fats, specifically TG, to be accumulated in the liver due to the excessive influx of free fatty acids from fat tissues, leading to lipotoxicity, mitochondrial impairment, and endoplasmic reticulum stress, which in turn can increase ROS production and inflammation, the two major triggers of NAFLD [41,55]. On the contrary, all polyphenols can attenuate TG accumulation in the hepatocytes by the regulation of lipogenesis, modulation of insulin resistance, controlling of inflammation, and modification of oxidative stress [56], which we found in groups fed AGs, indicating a potent antioxidant and hepatoprotective effect of AG plants due to the presence of many phenolic compounds that have antioxidant properties [57,58,59,60,61,62]; in addition, both vitamins C and E can function as scavengers of free radicals [63]. Polyphenols, in general, have been revealed to support liver therapy potential by boosting antioxidative defense enzymes via a number of different mechanisms including mediating nuclear factor erythroid 2-related factor 2 (Nrf2) expression and cytochrome P450 2E1 (CYP2E1) expression, alleviating inflammation by downregulating the activity of mitogen-activated protein kinases (MAPK)/nuclear factor kappa B (NF-kB) pathways, and downregulating apoptosis via the manipulation of B-cell lymphoma 2 (Bcl-2)/protein kinase B (AKT)/caspase expression [39]. Otherwise, this could also be due to the high-fiber nature of the AG plant whereby a high-fiber diet can be fermented by the gut microbiota with subsequent augmentation of gut microbiota-derived acetate, which in turn can regulate lipid metabolism during liver diseases [64].

Chronic feeding of a high-fat diet (HFD) to laboratory animals gives rise to hepatic steatosis and dyslipidemia through various pathways. Shortly following HFD feeding, free fatty acids stimulate the synthesis and accumulation of fat droplets within the liver [65]. In the present study, AG treatment lowered hepatic steatosis in the HFD rats compared to the controls, suggesting possible hepatoprotection via improvement in the lipid profile and fatty acid oxidation. AGs led to improved liver histology in the HFD groups. The effect of AGs on liver tissue has not been systematically evaluated previously. However, experimental studies on other Amaranthus species have been studied in animal models and showed significant improvements [66,67].

The potential of numerous plant-based compounds to treat NAFLD has previously been investigated. In both animal models and clinical investigations, green tea (*Camellia sinensis*), which contains the polyphenol epigallocatechin-3-gallate (EGCG), has demonstrated profound liver-protective effects. According to a systematic review report, green tea and its active compounds can enhance antioxidant defense, lower serum triglycerides and LDL cholesterol, and reduce fat accumulation in the liver by activating AMPK and reducing inflammatory markers such as TNF-α and IL-6 [68]. Our research findings are corroborated by an additional study, which determined that the phenolic extract of millet has an anti-hyperlipidemia and anti-steatotic effect on obese rodents [69]. Additionally, in another study conducted on obese rodents, the addition of green tea extract enhanced the body’s fat processing, reduced oxidative stress (as evidenced by increased levels of SOD, CAT, and GPx activity), and reduced liver injury, such as the MDA and ALT levels [70]. These findings lend credence to the notion that antioxidants and anti-inflammatory compounds derived from plants can enhance hepatic fat metabolism and mitigate liver damage. These observations are consistent with the results of our investigation, which employed *Amaranthus graecizans*. This clearly implies that the lipid-lowering and liver-protective effects observed may be attributed to the presence of bioactive components such as flavonoids and phenolic compounds.

Conclusively, the inference of this study is that isolated polyphenolic molecules have been reported to normalize the activity of antioxidative enzymes and to ameliorate histopathology by plummeting the inflammatory response by means of tempering oxidative stress, and likewise, by reducing oxidative stress after ischemia–reperfusion liver injury or obstructing hepatotoxicity-derived fibrosis growth in preclinical investigations through downregulating caspase-3, BCL-2, and NF-κB expression whereas it uplifted NRF-2-mediated mitochondrial functions. Likewise, ellagic acid and gallic acids are found in berries and fruits, have been reported to employ shielding effects in liver impairment caused by drug abuse via reducing TNF-mediated inflammation and lipid peroxidation. Alternatively, various plant-derived bioactive compounds may directly avert NAFLD and hepatic steatosis by lessening de novo lipogenesis via the direct downregulation of sterol regulatory element-binding protein 1c (SREBP-1c), increasing FA oxidation through upregulating PPAR receptor and improving insulin sensitivity, and decreasing intestinal lipid absorption.

Nevertheless, some limitations remain in this investigation. Herein, we used AG plants grown in the Al-Baha region; it would be very interesting to examine AG plants from other parts of Saudi Arabia. Furthermore, more well-designed research to investigate the molecular processes underlying these effects is strongly encouraged, such as targeting adipose and hepatic lipid regulators such as AMPK, SREBPs, and PPARs. Furthermore, our work emphasizes the importance of AG plant components as antioxidants, although we were unable to pursue this further. Consequently, it will be a fantastic topic for future research considering diverse parameters.

## 4. Materials and Methods

### 4.1. Chemical Reagents

Reagents used included ethanol (95%), petroleum ether, trifluoroacetic acid (0.05%), sodium carbonate, glucose, sulfuric acid (98%), sodium hydroxide, boric acid, hydrochloric acid (37%), and Tashiro’s indicator (pH 4.4–5.8), all obtained from Sigma-Aldrich (St. Louis, MO, USA).

### 4.2. Preparation of AG Powder

*Amaranthus graecizans* L. (AGs) was obtained from a local market in Al-Baha, Saudi Arabia. The plant was air-dried following Alibas [71], then ground into a fine powder using a 750-Watt Master Class Sanyo Mixer Grinder, Tokyo, Japan. The powder was stored in a clean, dry environment throughout the study.

### 4.3. Experimental Animals

Thirty male Wistar rats (150 ± 20 g, 10 weeks old) were housed under controlled conditions (22 ± 2 °C, 50% humidity, 12/12 h light/dark cycle) for a one-week acclimatization period with free access to food and water.

### 4.4. Diet Preparation

Control (SD) and high-fat diets (HFDs) were procured from Research Diets, New Brunswick, NJ, USA (Cat. No. D12450H and D12451, respectively). Table 5 details their nutritional composition.

### 4.5. Experimental Design

Rats were divided into five groups (*n* = 6 each): (1) SD + 0.25 mL 0.1% DMSO (control); (2) HFD + 0.25 mL 0.1% DMSO; (3–5) HFD + AG solution (0.25 mL) at 100, 200, or 500 mg/kg body weight, respectively, as per Zeashan et al. [72]. Treatments were administered daily via gavage for two months. Body weights were recorded weekly. After overnight fasting, rats were anesthetized (90 mg/kg ketamine, 10 mg/kg xylazine) [73], and 1 mL blood was collected via cardiac puncture, centrifuged (3000 rpm, 10 min), and serum stored at −20 °C. Livers were harvested post-euthanasia and preserved in 10% formalin.

### 4.6. Proximate Analysis

#### 4.6.1. Carbohydrates

Carbohydrate content was measured using the Hedge and Hofreiter [74] method. A 100 mg sample in a boiling tube was mixed with 5 mL of 2.5 N HCl and hydrolyzed in a water bath for 3 h. After cooling at room temperature, sodium carbonate was added until bubbling stopped. The solution was diluted to 100 mL with distilled water and centrifuged. Glucose standards of varying concentrations were prepared alongside. Aliquots (0.5 mL) of both sample and standards were adjusted to 1 mL with distilled water, mixed with 4 mL anthrone reagent, and heated in a boiling water bath for 8 min, shifted from green to dark green to indicate glucose. After rapid cooling, absorbance was read at 630 nm using a spectrophotometer. Carbohydrate content was determined from the glucose standard curve as follows:

Amount of carbohydrates present in 100 mg of the sample = (mg of glucose/volume of the test sample) × 100.

#### 4.6.2. Crude Protein

Crude protein was assessed using the Kjeldahl method [75], converting total nitrogen (N) to protein with a factor. For digestion, 5 g of the sample was placed in a flask with 20 mL concentrated sulfuric acid and a Kjeldahl catalyst (0.3% CuSO_4_·5H_2_O catalyst in 1000 mL solution). The mixture was heated at 350–380 °C for 3 h until partially whitened, then cooled and mixed with sodium hydroxide, turning blue to signal digestion completion. After adding 100 mL water, the solution was distilled with 50 mL 50% sodium hydroxide. Ammonia released was captured in 50 mL 4% boric acid with 6–7 drops of Tashiro’s indicator, shifting from reddish-violet to green (pH 4.4–5.8) as ammonium borate formed. This was titrated with 0.25 M HCl until a faint violet hue appeared. Nitrogen was calculated as follows:

% Nitrogen (N) = [(mL standard acid − mL blank) × N of acid × 1.4007/weight of sample (g)] × 100

% protein = N% × F (where F is the conversion factor equal to 6.25)

#### 4.6.3. Total Fat Content

Total fat content was measured using the hydrolysis method as per the AOAC Official Method 966.01 [76]. A 50 mL screw-top test tube with 1 g of AG powder was mixed with 1 mL ethanol, followed by 5 mL HCl. The mixture was heated to 75.5 °C for 40 min, shaken occasionally, then cooled to room temperature. Next, 5 mL ethanol, 12 mL anhydrous ether, and 12 mL petroleum ether were added sequentially, with shaking after each addition. After settling, the top ether layer was separated, transferred to a 150 mL beaker via a Pasteur pipette and filter paper, and repeated thrice with 8 mL ether each time. The extract was evaporated on a low-heat hot plate for 1 h, then heated at 135 °C for 10 min, cooled in a desiccator, and weighed. Fat percentage was calculated as follows:

% Fat = [(Weight of beaker and fat − tared beaker weight)/Sample weight)] × 100

#### 4.6.4. Moisture

Moisture content of AGs was assessed using ICC Standard No. 110/1 (1976) [77]. A pre-weighed, dry crucible with 2 g of sample was heated to 130–133 °C, cooled in a desiccator, and reweighed. Heating continued until a constant weight was achieved. Moisture percentage was calculated as follows:

*MC*% = (*w*2 − *w*1) − (*w*1 − *w*3) (*w*2 − *w*1) × 100%

w1 = weight of empty crucible. w2 = weight of crucible with sample. w3 = weight of crucible with dry sample.

#### 4.6.5. Ash

Ash content was determined following ICC Standard No. 104/1 (1990) [78]. A pre-weighed dry porcelain crucible with 2 g of sample was incinerated in a muffle furnace at 900 °C, for approximately 3 h until light gray ash formed. After cooling in a desiccator, the crucible was reweighed. Ash percentage was calculated using the formula:

*AC*% = [(*w*2 − *w*1)/*w*3] × 100%

AC = ash content. w1 = weight of empty crucible. w2 = weight of crucible after incineration. w3 = weight of the fresh sample

### 4.7. Micronutrient Analysis

#### 4.7.1. Vitamins

HPLC was conducted on an (Agilent 1260 system, Santa Clara, CA, USA) with an Eclipse C8 column (4.6 mm × 150 mm, 5 µm). The mobile phase, 0.01% TFA: methanol (70:30), flowed isocratically at 1 mL/min. A multi-wavelength detector monitored 248 nm. Samples (10 µL) were injected, and the column was kept at 40 °C.

#### 4.7.2. Minerals

Metal ion levels were measured using an Agilent 5100 SVDV ICP-OES (Serial No. MY15180008, Santa Clara, CA, USA). Dried samples (1 g) were digested in 250 mL Erlenmeyer flasks with 50 mL 20% HNO_3_, stirred, and heated at 70–85 °C for 48 h, with extra HNO_3_ added to maintain volume. Post-digestion, extracts were filtered (Nalgene, Thermo Scientific, Rochester, NY, USA), cooled, diluted to 100 mL with deionized water (Milli-Q, Ion Exchange Arabia, Jeddah, Saudi Arabia) and analyzed by ICP-OES. Glassware was rinsed thrice with 20% HNO_3_ and deionized water. Analysis used a PFA-ST/Peltier-cooled spray chamber at 1500 W RF power, with gas flow of 15 L/min (plasma), 1 L/min (nebulizer), and 0.83–0.88 L/min (auxiliary). The chamber was kept at 2 °C, pump speed at 0.5 mL/min, using nickel cones and dual lens/outlines detection. Scanning was in peak hopping mode (1 sweep/reading, 10 points/peak, 1 reading/replicate), performed in triplicate.

### 4.8. Phenolic Compounds

The phenolic compounds were analyzed using an Agilent 1260 series HPLC system (Santa Clara, CA, USA). Separation was achieved with a Zorbax Eclipse Plus C8 column (4.6 mm × 250 mm, 5 µm particle size). The mobile phase comprised water (A) and acetonitrile (B), both containing 0.05% trifluoroacetic acid, delivered at a flow rate of 0.9 mL/min. A linear gradient was applied as follows: 82% A from 0 to 18 min, maintained at 82% A from 18 to 24 min, reduced to 60% A from 11 to 18 min, then returned to 82% A at 0 min. Detection occurred at 280 nm using a multi-wavelength detector. Each sample was injected with 5 µL volume, and the column was kept at 40 °C.

### 4.9. Biochemical Analysis

Serum cholesterol levels were evaluated with a kit designed for rats (Cat. No. MBS168179, MyBioSource, San Diego, CA, USA). Serum triglyceride (TG) amounts were determined using a specialized rat assay (Cat. No. 10010303, Cayman Chemical, Ann Arbor, MI, USA). Levels of high-density lipoprotein cholesterol (HDL-c) and low-density lipoprotein cholesterol (LDL-c) were measured with colorimetric kits (Cat. No. K613-100, BioVision, Milpitas, CA, USA). Liver enzymes’ alanine aminotransferase (ALT) and aspartate aminotransferase (AST) were quantified with ELISA assays (Cat. No. MBS8801684 and MBS8804612, MyBioSource, San Diego, CA, USA). Activities of alkaline phosphatase (ALP) and gamma-glutamyl transferase (GGT) were assessed using Abcam kits (Cat. No. ab8336 and ab241029, Shanghai, China). Total bilirubin and protein concentrations were determined with kits (Cat. No. MBS730053 and MBS3808613, MyBioSource, San Diego, CA, USA). All steps adhered to supplier protocols, using samples from six rats per group.

### 4.10. Histopathology

Liver sections (4 µm) were fixed in 10% formalin, dehydrated, paraffin-embedded, stained with hematoxylin–eosin, and examined under a light microscope (Olympus, Tokyo, Japan) by an independent researcher.

### 4.11. Ethical Approval

The study was approved by Al-Baha University’s Research Ethics Committee (Ref. No. 46110115).

### 4.12. Statistical Analysis

Data were analyzed using GraphPad Prism 8 software with one-way ANOVA and Tukey’s post hoc test. Statistical significance was set at *p* < 0.05.

## 5. Conclusions

This study indicates that HFD triggers hyperlipidemia, liver damage, and fat accumulation in the liver. Administering AGs at different doses markedly reduced the serum levels of total cholesterol, TG, and LDL-C, while enhancing liver function and improving liver tissue structure. These results provide scientific support for AGs as an antioxidant and lipid-lowering agent in HFD-fed rats, mainly attributable to their rich polyphenolic compounds. Future research should prioritize human clinical trials to validate the safety and effectiveness of AGs for managing NAFLD. Additional studies exploring its mechanisms and optimal doses will strengthen its therapeutic potential. Moreover, AGs could gain traction as a natural remedy through the development of functional foods and the promotion of sustainable farming practices.

## Figures and Tables

**Table 1:** Proximate composition of *Amaranthus graecizans* L. on a dry weight basis (%).

Nutrients	%
Carbohydrates	31.5 ± 0.5
Protein	21.3 ± 0.3
Fat	1.1 ± 0.1
Total fiber	15 ± 0.1
Ash	25.6 ± 0.5
Moisture	5.15 ± 0.5

**Table 2:** Vitamin and mineral contents of *Amaranthus graecizans* L. on a dry weight basis.

Vitamins	µg/mL
Vitamin C (ascorbic acid)	8.09
Vitamin B1 (Thiamine)	7.16
Vitamin B3(Niacin)	1.39
Vitamin B9 (Folic acid)	6.37
Vitamin B12 (Cobalamin)	1.03
Vitamin A (Retinol)	4.69
Vitamin E (Tocopherol)	69.78
Vitamin D3(Cholecalciferol)	0.085
**Minerals**	**mg/g**
Magnesium (Mg)	6.55
Potassium (K)	35
Phosphorus (P)	2.5
Sodium (Na)	2.5
Calcium (Ca)	20

**Table 3:** Phenolic compounds of *Amaranthus graecizans* L. on a dry weight basis.

Compound Name	Formula	* RT (mine)	* MW (g/mol)	Peak Area (%)
Gallic acid	C_7_H_6_O_5_	3.557	170.12	16.96
Chlorogenic acid	C_16_H_18_O_9_	4.190	354.31	21.20
Catechin	C_15_H_14_O_6_	4.627	290.26	0.59
Methyl gallate	C_8_H_8_O_5_	5.471	184.147	2.47
Caffeic acid	C_9_H_8_O_4_	5.850	180.16	3.21
Syringic acid	C_9_H_10_O_5_	6.342	198.17	1.22
Rutin	C_27_H_30_O_16_	6.931	610.517	8.71
*p*-Coumaric acid	C_9_H_8_O_3_	8.522	164.04	2.69
Vanillin	C_8_H_8_O_3_	9.070	152.15	4.81
Ferulic acid	C_10_H_10_O_4_	9.642	194.18	2.03
Rosmarinic acid	C_18_H_16_O_8_	11.660	360.318	6.52
Daidzein	C_15_H_10_O_4_	15.658	254.23	0.54
Cinnamic acid	C_9_H_8_O_2_	19.162	148.1586	3.01

* RT, retention time; * MW, molecular weight.

**Table 4:** Effect of AGs on body weight gain in experimental rats.

Group	Body Weight Gain (g)
SD	9.83 ± 02.78
HFD	75.67 ± 11.79 ****
HFD + 100 mg/kg of AGs	49.00 ± 04.04 **^++++^**
HFD + 200 mg/kg of AGs	37.00 ± 09.03 **^++++^**
HFD + 500 mg/kg of AGs	39.00 ± 07.04 **^++++^**

Data are presented as mean ± SD (*n* = 6 rats/group). **** *p*-value < 0.0005; ^++++^ *p*-value < 0.0001 compared with (HFD) group.

**Table 5:** Energy in the diet of control and high-fat diet groups.

	SD (D12450H)		HFD (D12451)	
	gm%	Kcal%	gm%	Kcal%
Carbohydrate	67.3	70%	41	35%
Proteins	19.2	20%	24	20%
Fat	4.3	10%	24	45%
Others	9.7	-	11	-
Total (Kcal/gm)	100 (3.85 Kcal/g)		100 (4.73 Kcal/g)	

## Data Availability

The datasets used and analyzed during the current study are available from the corresponding author on reasonable request.

## Supplementary Materials

The following supporting information can be downloaded at: https://www.mdpi.com/article/10.3390/ph18081196/s1, File S1: HPLC profile of AGs plant.
