# Supplementary material for: Amaranthus graecizans L. Mitigates Hyperlipidemia-Induced Nonalcoholic Fatty Liver Disease in Experimental Rats: Future Pharmaceuticals
Source: Pharmaceuticals (Basel). 2025 Aug 13;18(8):1196. doi: 10.3390/ph18081196 (PMC12389473; doi:10.3390/ph18081196)
Supplement: Supplementary file 1 [file pharmaceuticals-18-01196-s001.zip › pharmaceuticals-3777098-supplementary.pdf]

Data File C:\CHEM32\1\DATA\PP 2-6-2024 2024-06-02 11-37-36\001-0201.D
Sample Name: Polyphenol STD

===

Acq. Operator	: SYSTEM	Seq. Line :	2
Acq. Instrument	: hplc -2	Location :	Vial 1
Injection Date	: 6/2/2024 12:04:48 PM	Inj :	1
		Inj Volume :	5.000 µl

Acq. Method : C:\CHEM32\1\DATA\PP 2-6-2024 2024-06-02 11-37-36\POLYPHENOL 2023.M
Last changed : 6/2/2024 11:37:39 AM by SYSTEM
Analysis Method : C:\CHEM32\1\DATA\PP 2-6-2024 2024-06-02 11-37-36\POLYPHENOL 2023.M (Sequence Method)
Last changed : 6/2/2024 2:38:02 PM by SYSTEM
(modified after loading)
Additional Info : Peak(s) manually integrated

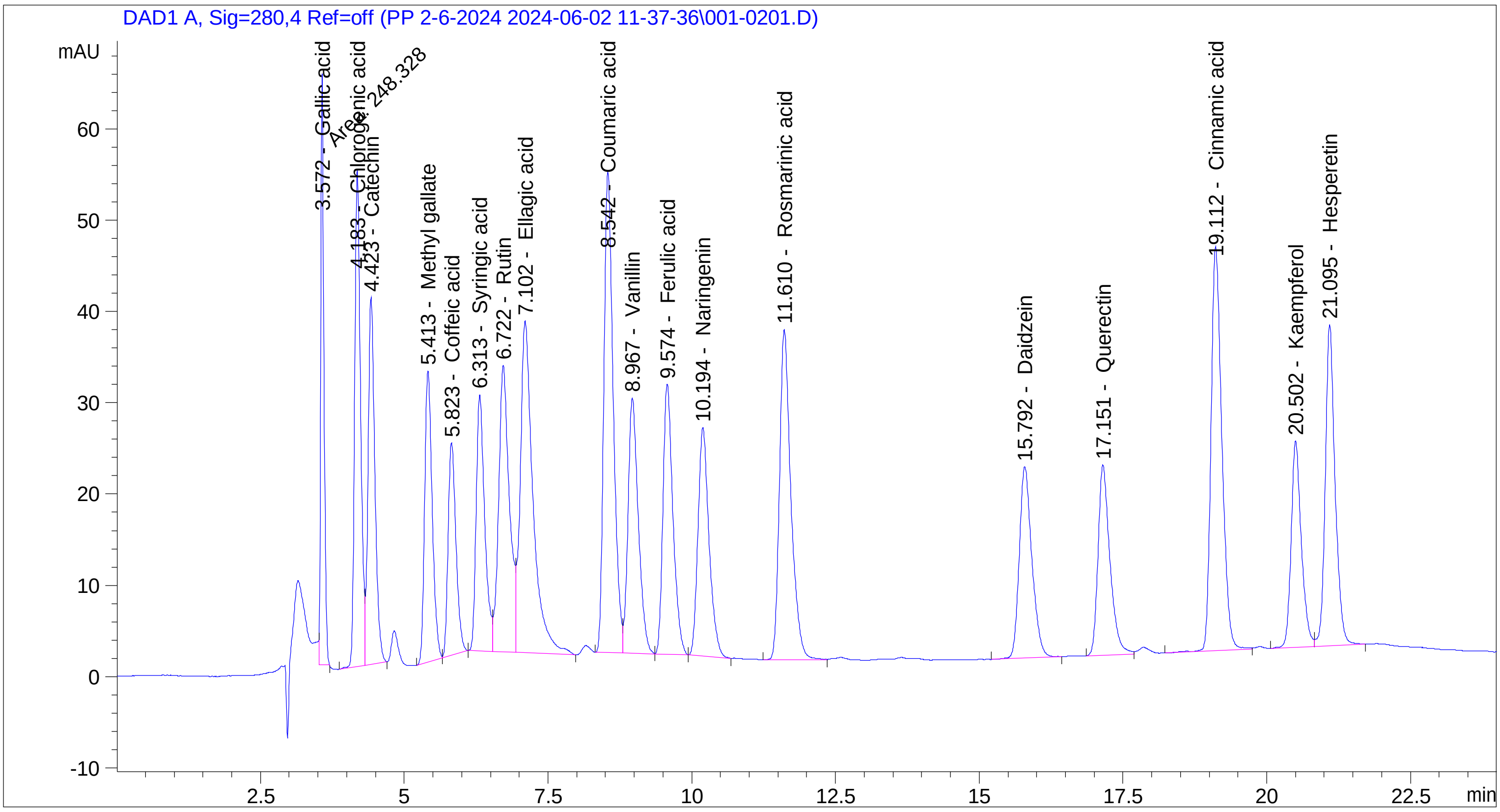

===

Area Percent Report

===

Sorted By : Signal
Calib. Data Modified : 6/2/2024 2:29:20 PM
Multiplier : 1.0000
Dilution : 1.0000
Do not use Multiplier & Dilution Factor with ISTDs

Signal 1: DAD1 A, Sig=280,4 Ref=off

Peak #	RetTime [min]	Type	Width [min]	Area [mAU*s]	Area %	Name
1	3.572	MM	0.0635	248.32758	3.6540	Gallic acid
2	4.183	BV	0.1078	391.71194	5.7638	Chlorogenic acid
3	4.423	VB	0.1216	329.14813	4.8432	Catechin
4	5.413	BV	0.1386	291.59103	4.2906	Methyl gallate
5	5.823	VB	0.1446	220.62531	3.2464	Coffeic acid

Peak #	RetTime [min]	Type	Width [min]	Area [mAU*s]	Area %	Name
6	6.313	BV	0.1544	289.68341	4.2625	Syringic acid
7	6.722	VV	0.1909	410.85287	6.0454	Rutin
8	7.102	VB	0.2264	568.31244	8.3624	Ellagic acid
9	8.542	BV	0.1729	600.84912	8.8411	Coumaric acid
10	8.967	VV	0.1879	350.03564	5.1506	Vanillin
11	9.574	VB	0.1828	357.32898	5.2579	Ferulic acid
12	10.194	BB	0.2018	334.35687	4.9199	Naringenin
13	11.610	BV	0.2056	501.19095	7.3747	Rosmarinic acid
14	15.792	BB	0.2300	319.64337	4.7034	Daidzein
15	17.151	BV	0.2119	300.41452	4.4204	Querectin
16	19.112	BV	0.1999	584.21869	8.5964	Cinnamic acid
17	20.502	BV	0.1821	279.09302	4.1067	Kaempferol
18	21.095	VB	0.1749	418.68427	6.1607	Hesperetin

Totals : 6796.06815

19 Warnings or Errors (10 first messages follow) :

Warning : Calibration warnings (see calibration table listing)
Warning : Invalid calibration curve, (Gallic acid)
Warning : Invalid calibration curve, (Chlorogenic acid)
Warning : Invalid calibration curve, (Catechin)
Warning : Invalid calibration curve, (Methyl gallate)
Warning : Invalid calibration curve, (Coffeic acid)
Warning : Invalid calibration curve, (Syringic acid)
Warning : Invalid calibration curve, (Rutin)
Warning : Invalid calibration curve, (Ellagic acid)
Warning : Invalid calibration curve, (Coumaric acid)

*** End of Report ***
